# Supplementary material for: Trnp1 organizes diverse nuclear membrane‐less compartments in neural stem cells
Source: EMBO J. 2020 Jul 6;39(16):e103373. doi: 10.15252/embj.2019103373 (PMC7429739; doi:10.15252/embj.2019103373)
Supplement: Supplementary file 1 — Expanded View Figures PDF [file EMBJ-39-e103373-s001.pdf]

# Expanded View Figures

**Figure EV1. Additional characterization of Trnp1 protein.**

A WB showing Trnp1 protein turnover. 24 hours after transfection with a plasmid expressing Trnp1-IRES-GFP, P19 cells were treated with cycloheximide (CHX) or CHX plus the proteasome inhibitor MG132. Cells were lysed at times indicated below the plot and immunoblotted for Trnp1 or GFP in WB. GFP (half-life: 24 h) was used as a loading control. The pixels for each band were measured and normalized to GFP so that the number of pixels at $t = 0$ was 100%. Mw: 35 kDa (blue line) 25 kDa (red line).

B Analysis of the coiled-coil structure of the Trnp1 (full line) and mutCCTrnp1 (dotted line) using the prediction tool COILS. The aa changes are represented in bold and underlined.

C Determination of the molecular mass of MBP-tagged Trnp1CC and mutTrnp1CC using size exclusion chromatography in combination with static light scattering. The refractive index (red for CC and blue for mutCC) signals were monitored and used to determine the molecular mass (black line). MBP-tagged Trnp1mutCC eluted as a monomer with a MW of 52.0 kDa. MBP-tagged Trnp1CC eluted in a broad peak with MW ranging from 0.5 to 2.0 MDa.

D, E Immunostaining for GFP (transfected cells), DAPI, and Trnp1 of P19 cells (D, E lower row) or E14 cortex cells (E, upper row) 24 h after transfection with plasmids expressing the different truncated (D) or mutated (E) forms of Trnp1-IRES-GFP indicated in the left panels. Note that the C-terminal truncated proteins are in the cytoplasm (D) and that the mutCC protein is nuclear in E14 cortex cells that have endogenous Trnp1 (E, upper row), but cytoplasmic in P19 cells lacking endogenous Trnp1 (E, lower row). Arrows show examples of Trnp1 localization in transfected (GFP+) cells.

F Immunostaining of Trnp1 and mutCCTrnp1 proteins expressed together with RFP in dissociated E14 cortical cells 24 h after co-transfection with plasmids expressing specific shRNA against Trnp1 (to knockdown the endogenous protein) or shcontrol plasmid with GFP (as in Stahl *et al*, 2013). Note that the mutCCTrnp1 protein loses its nuclear localization once the endogenous Trnp1 levels are reduced.

Data information: Scale bars: 10 μm.

© 2020 The Authors 

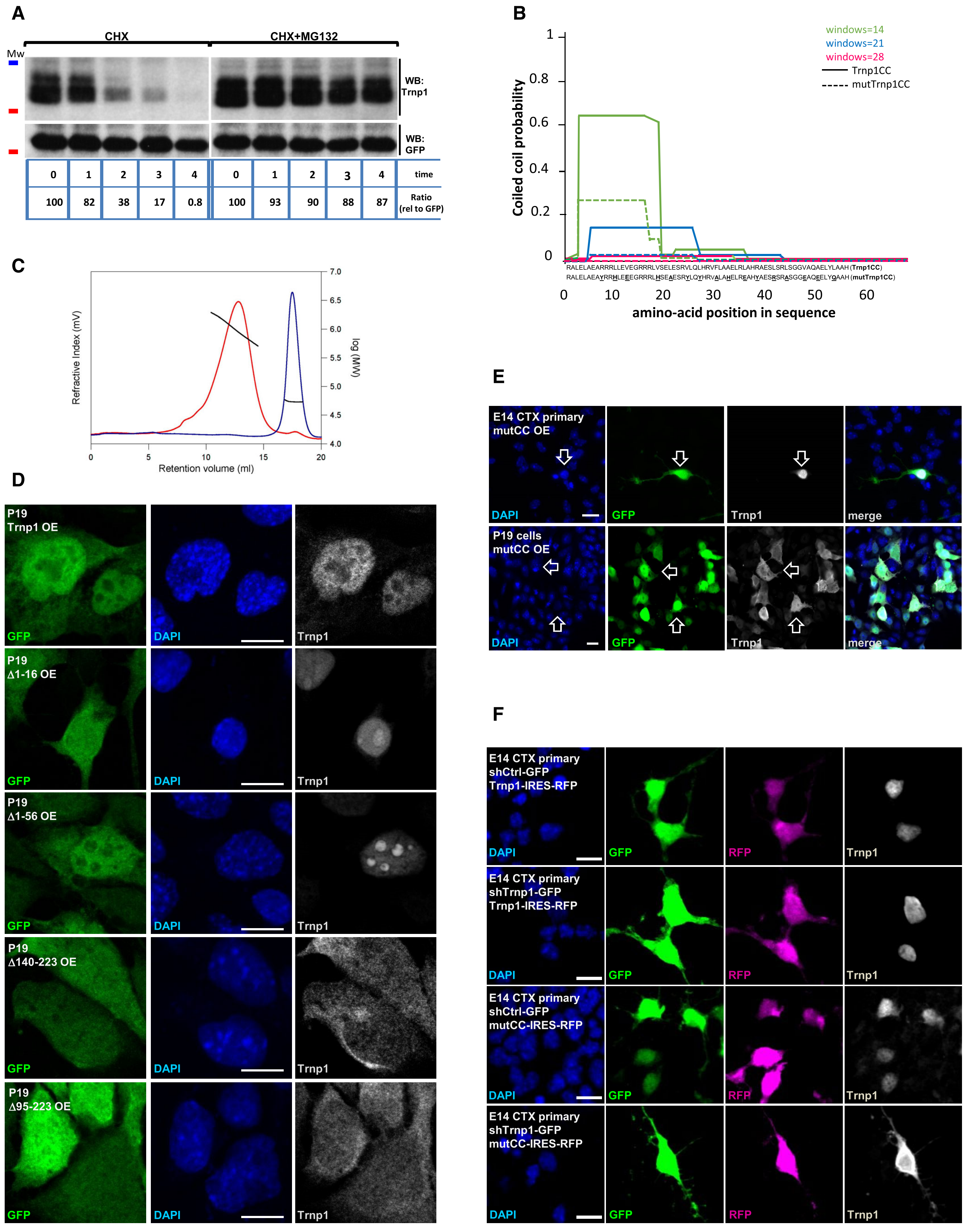


**Figure EV1.**

 © 2020 The Authors

**Figure EV2. N terminal FLAG does not affect Trnp1 localization, oligomerization capacity, turnover, or its function in promoting proliferation.**

A Immunostainings of Trnp1 and FLAGTrnp1 proteins 24 h after transfection in the indicated cells show nuclear localization for both proteins.

B Immunostaining of Ki67 and GFP in primary E14 cortical cells 24 h after transfection with plasmids expressing GFP, Trnp1-IRES-GFP, or FLAGTrnp1-IRES-GFP. Arrows show examples of double + cells (GFP, Ki67+ cells).

C, D WB showing FLAGTrnp1 protein turnover. 24 h after transfection, P19 cells were treated with cycloheximide (CHX) (C) or CHX plus the proteasome inhibitor MG132 (D). Cells were lysed at times indicated below the blot and processed for Trnp1 detection in WB. GFP (half-life:24 h) was used as a loading control. The pixels for each band were measured and normalized to GFP so that the number of pixels at $t = 0$ was 100%.

E Co-IP with anti-GFP and WB with anti-Trnp1 in lysates from P19 cells 24 h after transfection with plasmids expressing Trnp1-GFP fusion protein together with FLAGTrnp1-IRES-RFP. The control for the Trnp1-GFP is shown in Fig 1 as it is the same experiment.

F Top 10 ranked Gene Ontology terms and count of proteins that interact with Trnp1 analyzed by mass spectrometry shown in Fig 4B.

Data information: C–E are representative images of 3 independent experiments. Mw: 55 kDa (black line); 35 kDa (blue line); 25 kDa (red line). Scale bars: 10 μm.

© 2020 The Authors

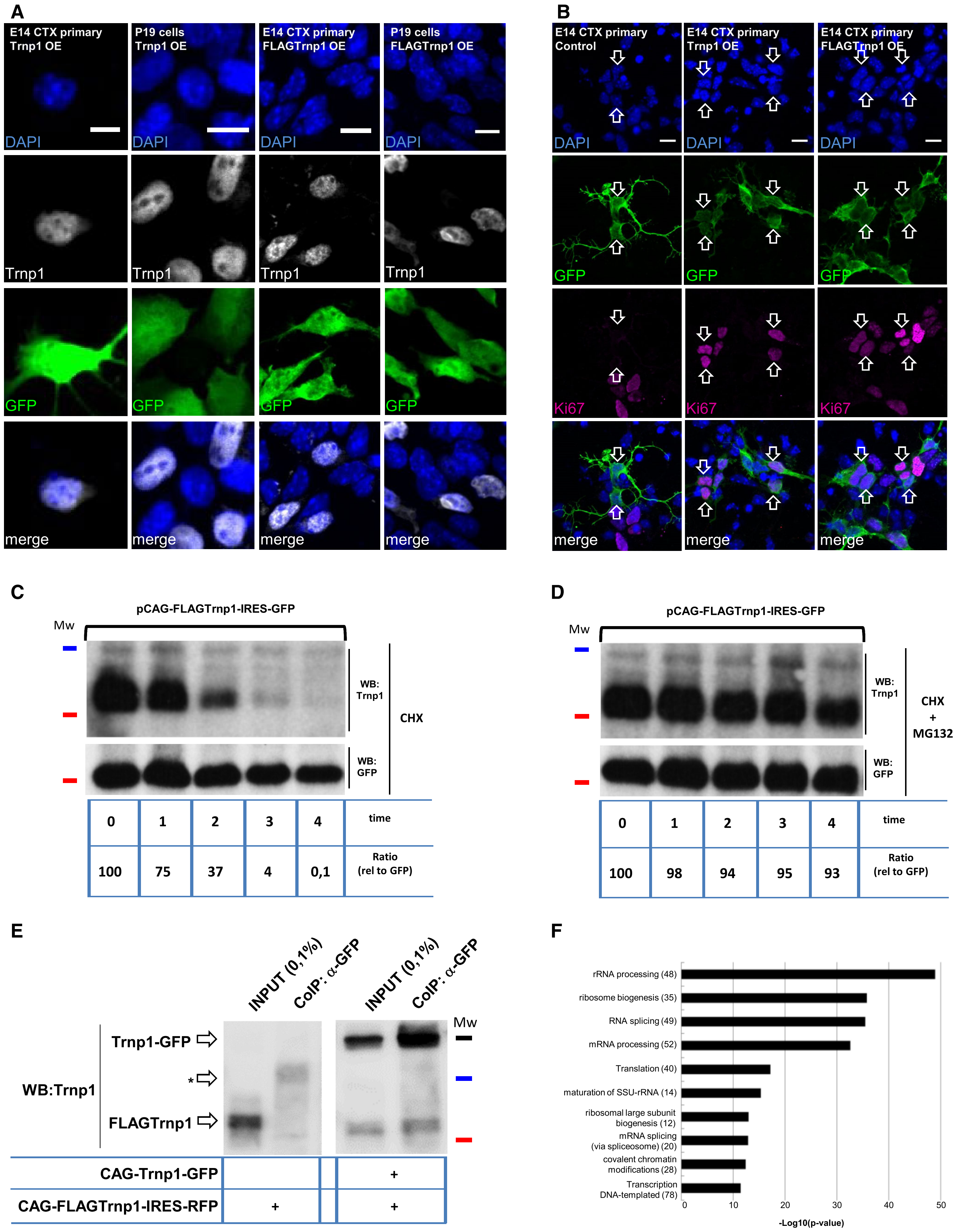


**Figure EV2.**

 © 2020 The Authors

**Figure EV3. Mutations of Trnp1 protein that fail to promote proliferation have a different cellular localization and cause mislocalization of some nucleolar and ribosomal proteins.**

A Micrographs showing GFP, DAPI, Ncl, and Trnp1 staining in P19 cells transfected for 24 h with plasmids expressing the indicated proteins together with GFP. The intensity line scan plot (right side; gray line for Trnp1, magenta line for Ncl and blue line for DAPI) measuring the gray value of each immunostaining as indicated was placed at the position of the yellow line in the micrographs. Note that Trnp1 peaks closely overlap with Ncl and its intensity decreases inside the nucleolus, while this is no longer the case for the mutant or truncated forms of Trnp1. C-terminal deletions lead to distribution of Trnp1 also outside the DAPI-high area, i.e., outside the nucleus. Scale bars: 10 μm.

B, C Micrographs showing GFP, DAPI, Fbl1, and FLAG (for Rpl26) staining in P19 cells transfected for 24 h with plasmids expressing the indicated proteins together with GFP and FLAG-Rpl26. Note that transfection with Trnp1 and mutCCTrnp1 shows a normal localization of Fbl1 and Rpl26, while the N-terminal deletion constructs of Trnp1 cause accumulation of Rpl26 around the nucleolus and a mislocalization of Fbl1. The C-terminal deletions of Trnp1 also show a normal localization of Rpl26 and Fbl1.

Data information: Scale bars: 5 μm. Yellow dotted shapes outline the nucleus to show examples of nucleolar and ribosomal proteins localization in transfected cells.

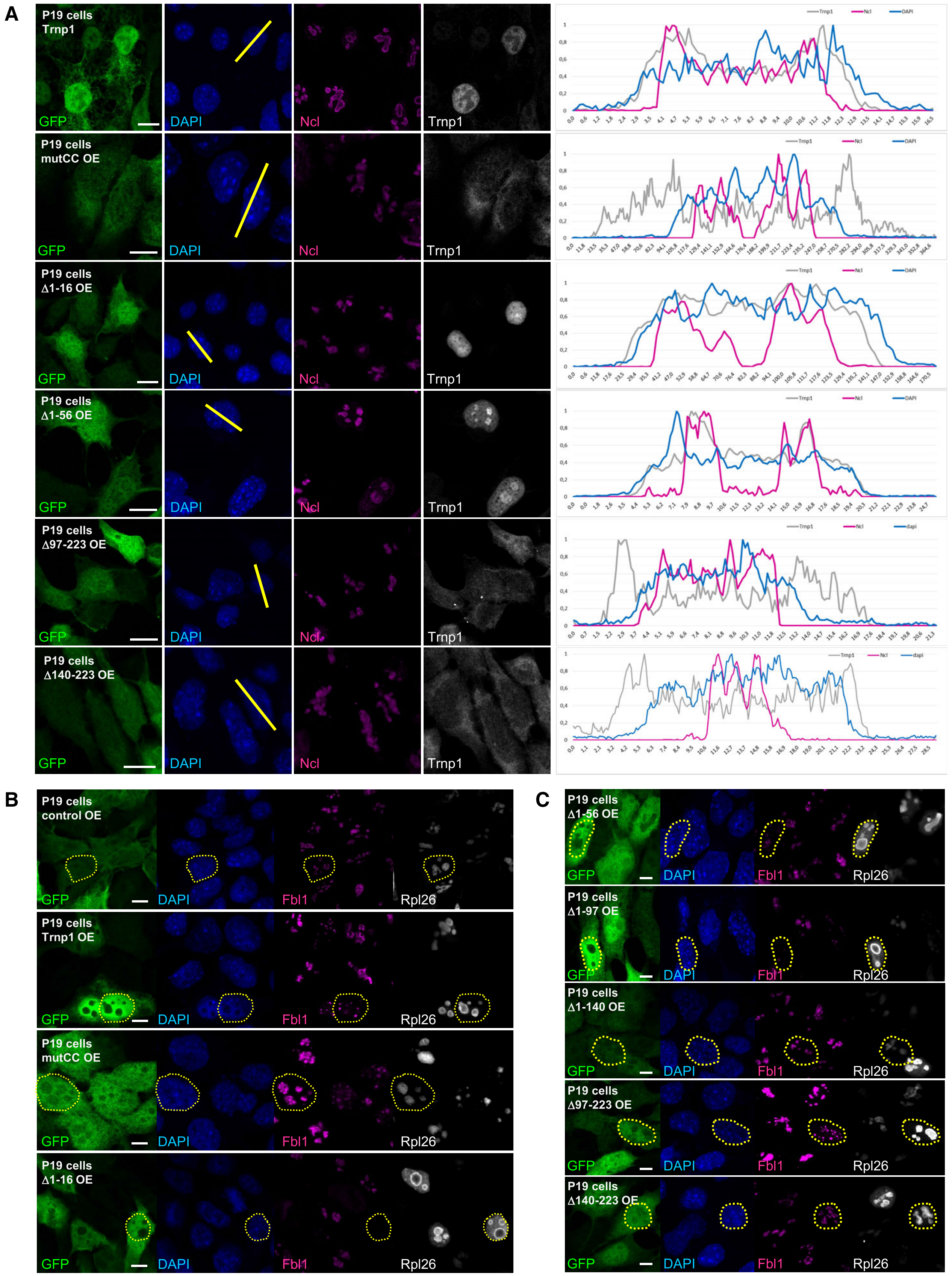


**Figure EV3.**

 © 2020 The Authors

**Figure EV4. Trnp1 expression in NSCs correlates with bigger nucleoli *in vivo*.**

A Micrographs of sections of E15 cerebral cortex showing DAPI (A) and immunostaining for Trnp1 (A′) and the nucleolar proteins B23 (for GC) (A″) and Fbl1 (for DFC) (A‴) with the boxed area shown in higher magnification to the right. Scale bars: 50 and 10 μm for magnifications.

B–D Micrographs of sections of E15 cerebral cortex in utero electroporated (IUE) 2 days earlier at E13 with the plasmids indicated showing DAPI and immunostaining for GFP (electroporated cells; B′, C′, D′), Pax6 (NSCs in the VZ; B″, C″, D″), and Tbr2 (TAPs; Non-VZ; B‴, C‴, D‴). Note that bipolar cells localized in the VZ are Pax6+, while multipolar cells localized in the Non-VZ are Tbr2+. Scale bars: 50 μm.

E Immunostaining for Trnp1 and DAPI in P19 cells transfected 24 h earlier showing nuclear puncta of Trnp1 in the nucleoplasm and accumulation of Trnp1 surrounding the condensed chromatin (green arrows). Scale bar: 10 μm.

F Representative FACS plots showing the isolation of the GFP+ cells 1 day post-electroporation (dpe) with shcontrol or shTrnp1 used for RNA-seq to determine alterations in alternative splicing.

Data information: V = ventricle; VZ: ventricular zone.

© 2020 The Authors

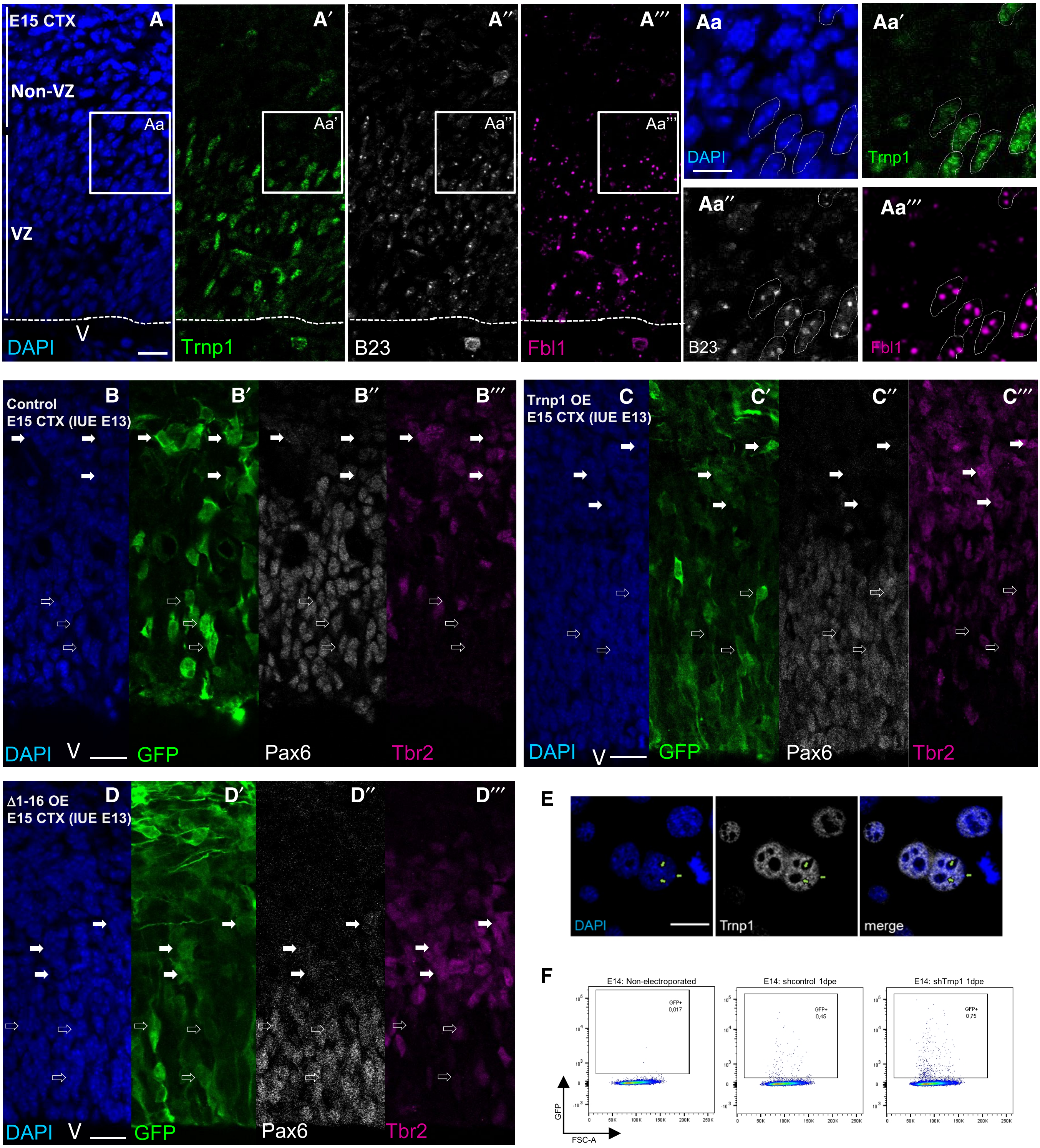


**Figure EV4.**



© 2020 The Authors
