# Supplementary material for: Trnp1 organizes diverse nuclear membrane‐less compartments in neural stem cells
Source: EMBO J. 2020 Jul 6;39(16):e103373. doi: 10.15252/embj.2019103373 (PMC7429739; doi:10.15252/embj.2019103373)
Supplement: Supplementary file 2 — Table EV1 [file EMBJ-39-e103373-s002.pdf]

Table EV1. List of antibodies used in this study.

Antibodies				
**target**	**conjugation**	**company**	**Cat no.**	**host**
Actin	-	Merck-Millipore	MAB1501	mouse
B23	-	Abcam	Ab10530	mouse
BrdU	-	Abcam	Ab6326	rat
Dhx15	-	Abcam	Ab70454	rabbit
Fibrillarin	-	Abcam	Ab5821	rabbit
GFP	-	Aves Lab	GFP-1020	chicken
GFP	-	eBioscience	14-6774-81	rabbit
Hist1.5	-	Abcam	Ab18208	rabbit
Hist3	-	Abcam	Ab1791	rabbit
HnRNP M3 M4	-	Abcam	Ab9548	mouse
HSPA8	-	Enzo	ADI-SPA-815	mouse
Ki67	-	Abcam	ab92742	rabbit
Ki67	-	ThermoFischer	14-5698-82	rat
M2-FLAG		Sigma	M3165	mouse
Matr3	-	Abcam	Ab84422	rabbit
Mlf2	-	Abcam	Ab201320	rabbit
Nacc1	-	Abcam	Ab29047	rabbit
Nap1L1	-	Abcam	Ab33076	rabbit
Nucleolin	-	NEB	14574S	rabbit
Pax6	-	Millipore	AB2237	rabbit
Pax6	-	Abcam	78545	mouse
pHH3	-	Millipore	06570	rabbit
Pnn	-	Bethyl lab	A301-022A	rabbit
RFP	-	Rockland	600401379	rabbit
Supt16H	-	Abcam	Ab204343	rabbit
Tbr2	-	Merck-Millipore	ab2283	rabbit
Trnp1		Homemade (Stahl et al., 2013)		Guinea pig
mouse IgG	HRP	Ge Healthcare Life Sciences	NA931-1ML	sheep
rat IgG	HRP	Ge Healthcare Life Sciences	NA935 (VWR International)	goat
rabbit IgG	HRP	Ge Healthcare Life Sciences	NA934-1ML	donkey
Chicken IgG	HRP	Abcam	ab97135-1mg	-
Guinea pig IgG	HRP	Santa Cruz	sc-2903	-
chicken	A488	ThermoFischer	A11039	-
rat	A488	ThermoFischer	A11006	-
rat	A546	ThermoFischer	A11081	-
rat	A647	ThermoFischer	A21247	-
rabbit	A488	ThermoFischer	A11008	-

rabbit	A546	ThermoFischer	A11010	-
rabbit	A633	ThermoFischer	A21070	-
rabbit	A647	ThermoFischer	A21244	-
guinea pig	A647	ThermoFischer	A21450	-
mouse IgG	A488	ThermoFischer	A11034	-
mouse IgG	A546	ThermoFischer	A11033	-
mouse IgG	A647	ThermoFischer	A32728	-
Guinea pig IgG	Abberior STAR 635P	Abberior	ST635P-1006	-
