# Supplementary material for: Trnp1 organizes diverse nuclear membrane‐less compartments in neural stem cells
Source: EMBO J. 2020 Jul 6;39(16):e103373. doi: 10.15252/embj.2019103373 (PMC7429739; doi:10.15252/embj.2019103373)
Supplement: Supplementary file 3 — Table EV2 [file EMBJ-39-e103373-s003.pdf]

Table EV2. List of primers used in this study.

Primers	
**name**	**Sequence (restriction sites bold)**
N-flag-TRNP1-forw	TCCGCATCT**GAATTC**CCCACGCACATGGATTACAAGGACGACGATGATAAGGGGATCCCGGGCTGCCGAA
N-flag-TRNP1-rev	GGGTCTAGATAT**CTCGAG**GGATACTC
Delta 1-16 forward:	CCCAG**GTCGAC**ATGGCAGAACCGGGGTCGCCCCC
Delta 1-56 forward:	GCCCT**GTCGAC**ATGGCCGAGACACCCGTGGAAGGG
Delta 1-97 forward:	GGCAG**GTCGAC**ATGGAGCTGGCCGAAGCGCGG
Delta 1-140 forward:	GCCTG**GTCGAC**ATGGCCGAGAGCCTGAGCCGC
Delta 56-223 reverse:	GCACC**CTCGAG**TTAGCGCTGCAGCTCCTG
Delta 95-223 reverse:	TATTA**CTCGAG**CTAACGGCCCCCTGCCCCC
Delta 140-223 reverse:	CTTGA**CTCGAG**ACCCGTGCTAAGCCAGGTAGAGCTC
Trnp1Aggreg Forw 1	GAAGCGC**GTCGAC**GACTGCTAGAAGTGGAGGGC
Trnp1Aggreg Forw 2	GCGCCT**GTCGAC**TCGCGCCGAGAGCC
Trnp1Aggreg Forw 3	GCGCACGG**GTCGAC**TCTCAAGAAGGGTGCGC
Trnp1Aggreg Rev 1	GCCGC**GGATCC**GGCTCAGGCTCTCGGCG
Trnp1Aggreg Rev 2	ACTCC**GGGATCC**GCGGCGCGCACC
Trnp1Aggreg Rev 3	CTCC**GGATCC**TGGCCGCGCCGTA
recombTrnp1CC forw	GGCGAAGG**CCATGG**GCCGTGCGCTGGAGCT
recombTrnp1CC rev	CACCCTT**CTCGAG**ACGGGATCAGTGCGCA
qPCR Trnp1-forw	AGTCAGCTGGGGGTCCAT
qPCR Trnp1-rev	ATGCAGAAGTCAGTCCAGACC
Oligo mutCC Trnp1	ATCCGGATATAGTTCCTCCTTTCAGCAAAAAACCCCTCAAGACCCGTTTAGAGGCCCCAAGGGGTTATGCT<br>AGTTATTGCTCAGCGGTGGCAGCAGCCAACTCAGCTTCCTTTCGGGCTTTGTTAGCAGCCGGATCTCAGTG<br>GTGGTGGTGGTGGTG**CTCGAG**TCAGCGCTGGGGGGAGGCGGGGCCGCGGGGCGGGCTGCGGCGGAAGGG<br>CGAGTCGGGCTCCGGGCCCTGGCCGCGCCGTAGCCGCCCTGCGCCCCAGGGCACGCAGCTCCCCAGGCCGA<br>GCGCGGACGCCAGCAGCGCCGGGGGACGACCCCGGCGACCGCGGCGCGCACCCTTCTTGAGACGGGACCC<br>GTGCGCAGCCTGGTAGAGCTCCTCCTGGGCCTCGCCGCCGCTCGCGCGGCTGCGGCTCTCGGCGTAATGCG<br>CCTCGCGCAGCTCATGAGCCAATGCGACTCGGTGGTACTGCAGGTACCGGCTCTCCGCCTCCGAATGCAGG<br>CGCCGGCGGCCCTCCTCTTCTAGCAGATGCCGCCGGTATTCGGCCAGCTCCAGCGCACGGCCCCCTGCCCCC<br>GCCGCCGCGCCCGCGATCCCCGCCGGGCCCGCGCCCCCAGACCCCCCACTAGCACCCTGGCGCCAGCGCTG<br>CAGCTCCTGCCCTTCCACGGGTGTCTCGGCCGCCTCGGGTAAGGGCGGTGACTTAGTGGGGGTCGGAGTCG<br>AGGTCGGCGATGGGGAAGGGGGCTGGAGGGACGGCAGGGGCTCCCGGGGTGGCGGCGGGGGCGACCCCG<br>GTTCTGCCGTCCCTTCCTGGGCCCCTGGGCCGCAGGCGCTGATTCGGCAGCCCGGCATCG**CCATGG**CGCC<br>**GGATCC**ATAAAGATTCTCAGAACCACTGCCAGATCCCGAATTAGTCTGCGCGTCTTTCAGGGCTTCATCG<br>ACAGTCTGACGACCGCTGGCGGCGTTGATCACCGCAGTAC
